# A Passing Notice of the Therapeutic Application of Psychology

**Published:** 1900-10

**Authors:** Willis B. Parks

**Affiliations:** Atlanta, Ga.


ATLANTA

### Journal-Record of Medicine.

**Successor to Atlanta Medical and Surgical Journal, Established 1855, and Southern Medical Record, Established 1870.**

VOL. II. OCTOBER, 1900. No. 7.

EDITORS { BERNARD WOLFF, M.D., DUNBAR ROY, A.B., M.D.

M. B. HUTCHINS, M.D., BUSINESS MANAGER.

**Published Monthly. Office No. 64 Marietta Street, Opposite Post-office.**

### *ORIGINAL COMMUNICATIONS.*

---

### A PASSING NOTICE OF THE THERAPEUTIC APPLICATION OF PSYCHOLOGY.

BY WILLIS B. PARKS, M.D.,

ATLANTA, GA.

We might say at this advanced time that we are soaring on the wings of progress; the "wings" are tipped with "faddism," and after the shining luster of the tips are worn off, from beating hard against reason and actual experiments, minus theory, we may expect to still soar on the wings of progress not having been worsted by the faddisms, but rather the better "for the wear."

In presenting this subject of therapeutic psychology, I am aware that the medical profession have been more or less disgusted, and, I may say, discouraged, from properly investigating this subject on account of the element of unscrupulous quackery and charlatanry in advertising unreasonable results by psychological methods in the treatment of nearly all diseases to which mankind is heir. Yet all this should not deter us from an earnest scientific investigation of methods and means that might alleviate some of the many ailments of our patients, for if Lister had even hesitated at mock derision, antiseptic surgery might not have been developed and perfected as it is to-day; if Massey had left electricity in the hands of the quack, we would not have known its valuable uses by electrolysis and cataphoresis; so, too, serum-therapy would have fallen in the face of severe criticism, and thus it has been all along the line of improvement of every department of progressive medicine.

Therapeutic psychology stands to-day at the threshold of the medical profession, waiting for a legitimate and honest investigation.

I will presume to show that some progress has been made, especially since Hudson has introduced his work on "New Psychology," a work which has greatly simplified the subject, relieving very much the mysterious and the metaphysical and unknowable something that has seemingly been shrouded in mystery as taught in the old empirical psychology.

In order to simplify the "New Psychology," as taught by Hudson, we will first view it synthetically. Admitting the duality of the mind, the one he calls the objective mind, the other he calls the subjective mind; or, from an anatomical standpoint, we may call the first voluntary mind, and the other we call automatic mind. The objective, or voluntary, mind includes all the reasoning faculties and all conscious perceptions that come through the five senses. The subjective or automatic mind is that mysterious something that physicians usually call "vis-medicatrix-naturæ." Hence, we have the synonymous terms subjective, automatic, involuntary and vis-medicatrix-naturæ, meaning one and the same thing, which is the part of the duality of the mind that we are supposed to impress or persuade or stimulate when medicines are given, or remedial agents are applied to our patients. All physicians will readily acknowledge that the desired effects of medicines given, and agents applied, depend greatly on the confidence the patient has in the physician and the medicines. This word, or term, "confidence," we will beg leave to define nothing more than "suggestion," granting that the privilege is given to change the term "confidence" to "suggestion." Then all physicians are dependent and constantly using "suggestion" to stimulate and reinforce the automatic mind of their patients in the relief of their many ailments, and we will presume to say that doubtless suggestion often takes the lead, as proven in the happy effect the astute physician often gets when he confidently administers his "placebo."

In summing up the synthetical part, if objections are made to the duality of the brain or mind, we will concede that there is one brain with two sets of functions, viz.: automatic and voluntary, which amounts to the same as duality.

In presenting the analytical part of the subject, we will, for the benefit of those physicians who are materialists, offer a few points of anatomy now accepted as true. Mills, in his recent work on the Nervous System, says: "It has usually been taught that the nerve cells and nerve fibers are different structurally. Strictly speaking, this is not true. They are," he says, "parts of the same histological unit, as has been shown by recent investigation." By this he means that the nerve cell and the nerve fiber are one entity, the neuron. "Each neuron is a single entity with physiological connection only with the other cell or neuron." Now, according to Schafer's Cellular Terminology, each neuron has two sets of processes: the one axis-cylinder called neuraxon, and the other processes are protoplastic. He calls them dendrons. It has been shown that the protoplastic dendrons convey impulses toward the cell, which corresponds to voluntary life; the axis-cylinder, or neuraxon, convey impulses away from the cell, which corresponds to automatic life.

It will appear from the above facts in regard to cell structure of the nerve, also with its cellular terminology, that we also have the duality of the brain or mind, as Hudson teaches in his "New Psychology," but we have the same corresponding voluntary and automatic feature through all the nervous system. Hence, it will be seen that the object of so elucidating this subject of therapeutic psychology is to maintain it and apply it to the undisputed rules and facts of anatomy and physiology, where all therapeutic agents have their origin and application; then we will be permitted, without violating our preconceived ideas of the imaginary line drawn between the physical and the metaphysical, to use or apply psychology in the practice of medicine when indicated.

For those who are skeptical as to the efficacy of psychological suggestion in the practice of medicine, I will attempt to explain the *modus operandi* of this valuable agent as it is used by those who employ it intentionally, as also the same laws are used by those who get the benefit of it unintentionally, the latter, though, at a great disadvantage for lack of the necessary technique.

In order to make matters clear, we will classify the cerebellum, basal ganglion and the spinal cord as the automatic or involuntary mind. The cerebrum represents the voluntary mind. Now, if we wish to get a subject into the proper condition to receive "suggestion," we will cause fixation of the eyes of the patient to be treated on some indifferent point. This is the first step to render him passive, because impressions from the eyes go first to the basal ganglia, thence to the voluntary brain center. If we wish sleep to occur we will prolong the fixation of the eyes, which increases the stimulation of the basal ganglia, and as it is connected with the cerebellum and the spinal cord, which includes all the automatic machinery, all of which will be stimulated and become dominant and ready for any suggestion we make, provided life and liberty are not called into question.

As all growth, nourishment and repair, from the least cell to the mightiest organ or tissue, is dependent on the automatic brain, is it any wonder when strong suggestions are brought to bear on the automatic brain, when the voluntary brain is passive, that we control many neurotic conditions that have their origin from a perverted condition of both automatic and voluntary life?

It will be remembered that it was intimated in the first part of the subject that we are dependent on the proper stimulating of the automatic brain to get the desired results, whether it is done through medicines, electricity, etc., or by "suggestion."

